# Esthetic Perception of Gingival Display by Laypeople and Diagnostic Impressions and Etiologic Attribution of Gummy Smile by Dental Professionals: A Cross‐Sectional Study

**DOI:** 10.1155/ijod/4499170

**Published:** 2026-08-04

**Authors:** Nimet Turan, Priti Charde, Esra Guzeldemir-Akcakanat

**Affiliations:** ^1^ Private Practice, Kocaeli, Türkiye; ^2^ College of Dental Medicine, QU Health, Qatar University, Doha, Qatar, qu.edu.qa

**Keywords:** gingival display, gummy smile, smile esthetics, smile line, visual analog scale

## Abstract

**Objective:**

This study evaluated the diagnostic impressions of gummy smiles and perceived etiologic attribution among general practitioner (GP) dentists and dental specialists and compared the esthetic perception of gingival display between dental professionals and laypeople.

**Materials and Methods:**

Twelve digitally modified smile photographs were generated from a master photograph by altering gingival margin levels and lip position using Adobe Photoshop. A questionnaire was administered to five groups: GP dentists, periodontists, orthodontists, prosthodontists, and laypeople (50 per group; *n* = 250). Participants rated each image using a 10‐cm visual analog scale (VAS). Intergroup comparisons used the Kruskal–Wallis test with Bonferroni‐adjusted Mann–Whitney *U* tests; categorical variables were analyzed using the chi‐square and Fisher–Freeman–Halton (FFH) exact tests.

**Results:**

Significant intergroup differences in esthetic perception were observed for most photographs (*p*  < 0.05). Orthodontists consistently assigned lower esthetic scores than all other groups and were more likely to diagnose gummy smile. Laypeople rated images with mild‐to‐moderate gingival display more favorably. Orthodontists more frequently attributed gummy smiles to skeletal etiology; periodontists and GPs favored soft tissue‐related causes.

**Conclusions:**

Esthetic perception of gingival display varies significantly among dental specialists and between professionals and laypeople. Differences were also observed in the diagnostic impressions and perceived etiologic attribution of gummy smiles among dental specialties. These findings highlight the subjective nature of gummy smile assessment and emphasize the importance of considering both professional evaluation and patient perception during esthetic treatment planning.

**Clinical Significance:**

The findings of this study demonstrate that the esthetic perception of gingival display, diagnostic impressions of gummy smile, and perceived etiologic attribution may vary between dental professionals and laypeople. Awareness of these differences may facilitate interdisciplinary communication and support a more comprehensive and patient‐centered approach to the assessment of gummy smiles.

## 1. Introduction

The smile is one of the primary determinants of facial attractiveness and plays a central role in nonverbal communication. It significantly influences self‐confidence and social perceptions [1]. Because the oral region is central to facial expression, the smile contributes substantially to perceived attractiveness [2, 3].

In recent years, particularly with the influence of social media, facial and smile esthetics have gained increasing importance across all age groups. Although perceptions of beauty may evolve over time, smile attractiveness remains closely linked to overall facial harmony. For example, while flatter profiles were considered attractive in the past, today profiles with fuller and more pronounced lips are seen as more attractive [4, 5].

Smile esthetics require a balance among dental and gingival components. Sabri [6] identified eight key elements of a balanced smile: smile line, smile arc, upper lip curvature, buccal corridors, frontal occlusal plane, smile symmetry, and dental and gingival components. Among these, gingival display plays a crucial esthetic and functional role.

The literature reports varying opinions regarding acceptable gingival display. Some studies suggest 0–2 mm as ideal [7–9], while others consider up to 4 mm acceptable [5, 10]. Gingival exposure exceeding 3–4 mm is generally classified as a gummy smile [11]. Despite these definitions, esthetic perception may vary between professionals and laypeople [12].

Previous studies have evaluated multiple smile parameters simultaneously using different tools such as two‐dimensional photographs, three‐dimensional images, and dynamic video recordings [5, 10, 13–18]. Because smiling is a dynamic facial expression, several authors have suggested that dynamic records may provide diagnostic information that cannot be fully captured by static images [19, 20]. However, fewer investigations have focused specifically on gingival display. Moreover, comparative evaluations among different dental specialties and laypeople remain limited. We hypothesized that dental specialists would demonstrate greater sensitivity to gingival display variations compared to laypeople and that etiology perception would differ across specialties. Therefore, the present study aimed to assess how different dental professionals diagnose gummy smiles and to compare their esthetic perceptions with those of laypeople.

## 2. Materials and Methods

This cross‐sectional observational study was approved by the Ethics Committee of the Medical Faculty of Kocaeli University (Approval Number GOKAEK‐2024/08.10; 08.05.2024). All participants received written and verbal information about the study and provided informed consent before participation.

### 2.1. Sample Size

Based on a G^∗^ Power analysis (type I error *α* = 0.05, power 1‐*β* = 0.80), the minimum required sample size was determined to be 38 participants per group. Considering a potential 10% attrition allowance, at least 42 participants per group (total 210) were deemed necessary to achieve adequate statistical power. To further strengthen the study design and minimize the risk of reduced power due to potential data loss, 50 participants were ultimately recruited for each group, resulting in a total sample size of 250.

### 2.2. Image Acquisition and Preparation

To eliminate potential bias related to facial hair (e.g., beard or moustache), which may influence the esthetic perception, all study photographs were obtained from female subjects. Inclusion criteria were no previous orthodontic treatment, absence of dental anomalies, no active periodontal disease, no anterior restorations, and no history of treatment affecting gingival esthetics or smile appearance.

Frontal smile photographs were captured from six volunteer female individuals exhibiting smile characteristics close to established esthetic norms. All images were taken against a plain black background, with participants in a natural head position, at a standardized distance of 1.5 m, using a digital camera (Nikon D7100; Nikon Corporation, Tokyo, Japan) equipped with a 105‐mm macro lens (AF‐S Micro Nikkor 105 mm; Nikon Corporation). Static photographs were used to ensure standardized evaluation conditions, to allow precise manipulation of gingival display levels, and to minimize potential confounding factors associated with interindividual anatomical variations. Characteristics such as lip morphology, tooth shape, gingival architecture, and facial proportions may independently influence the esthetic perception and potentially obscure the specific effect of gingival display on smile attractiveness.

The six standardized smile photographs were independently evaluated by 10 graduate residents in periodontology according to their overall subjective esthetic perception, and the photograph selected by consensus among the evaluators was chosen as the master (reference) photograph. The master photograph was selected to provide a standardized and esthetically balanced reference image for subsequent digital modifications. It belonged to a young adult female with harmonious smile characteristics and no apparent dental anomalies.

All images were processed using Adobe Photoshop CC 2024 (Adobe Systems, San Jose, CA, USA). The selected photograph was cropped to include only the lips, gingiva, and teeth, excluding the nose, chin, cheeks, eyes, and forehead to minimize confounding facial influences on the esthetic perception.

**Figure 1:**
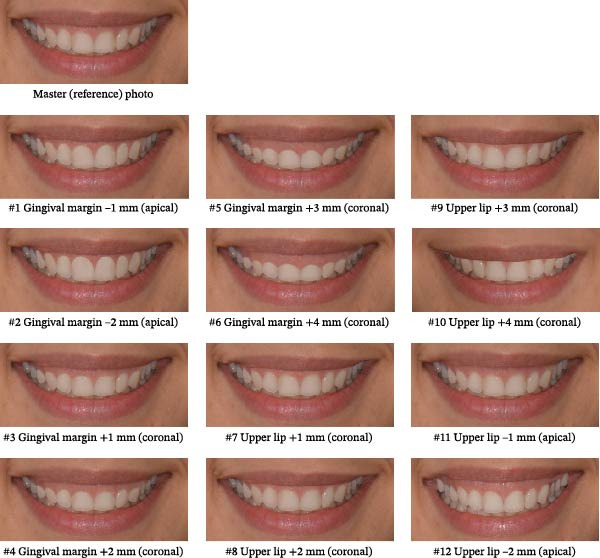
Master (reference) photograph and digitally modified images showing variations in gingival display. Images #1–#6 were created by altering the gingival margin position, whereas images #7–#12 were generated by modifying the upper lip position. The magnitude and direction of each modification are expressed relative to the reference image.

The master and modified photographs are shown in Figure 1, and the characteristics of each modification are summarized in Table S1. The gingival display was digitally altered by vertically adjusting the gingival margin by −1 mm apically (Photo 1), −2 mm apically (Photo 2), +1 mm coronally (Photo 3), +2 mm coronally (Photo 4), +3 mm coronally (Photo 5), and +4 mm coronally (Photo 6). The upper lip position was subsequently modified using the same incremental values as follows: the upper lip line was shifted +1 mm upward (Photo 7), +2 mm upward (Photo 8), +3 mm upward (Photo 9), +4 mm upward (Photo 10), −1 mm downward (Photo 11), and −2 mm downward (Photo 12), yielding a total of 12 modified images. For clarity, photographs #1–#6 represent variations in the gingival margin position (ranging from −2 mm apically to +4 mm coronally relative to the reference), while photographs #7–#12 represent modifications in the upper lip position (ranging from −2 mm inferiorly to +4 mm superiorly relative to the reference). The reference (master) photograph represents the unmodified baseline image.

### 2.3. Participants

The selected dental professionals (periodontics, orthodontics, prosthodontics, and general dentistry) represent the disciplines most commonly involved in the diagnosis and management of gummy smiles. Licensed general practitioner (GP) dentists and specialists in periodontics, orthodontics, and prosthodontics, as well as laypeople who voluntarily agreed to participate, were included in the study. These professionals frequently evaluate the gingival display and smile esthetics in clinical practice.

All interviews were conducted face‐to‐face by a single member of the research team to ensure standardization. All participants evaluated the photographs under identical conditions. The photographs were presented individually in a digital format in the same predefined order for all participants under standardized viewing conditions. Participants were allowed to review each image without time restriction before recording their responses.

For laypeople, the inclusion criteria were as follows: age ≥18 years; absence of visual impairment that could affect image evaluation; no professional education or training in dentistry or related fields; no history or experience related to orthodontic treatment or oral and dental anomalies; no self‐reported active periodontal disease; no anterior dental restorations or previous treatments affecting gingival esthetics or smile appearance; and self‐reported perception of good oral hygiene and an overall healthy dental appearance.

Exclusion criteria included age under 18 years, impaired mental health, visual impairments affecting image assessment, and failure to provide voluntary informed consent.

### 2.4. Questionnaire Design and Study Procedure

Two separate questionnaire forms were developed for this study: one for dental professionals and one for laypeople.

The questionnaire administered to dental professionals consisted of two sections. The first section collected demographic and professional background information through 11 items, including gender (female/male), age, dental specialty (GP dentist, periodontist, orthodontist, or prosthodontist), and years of professional experience in the dental practice. Participants were also asked whether they had previously received orthodontic treatment and whether they had ever been treated for a gummy smile. Additional items assessed their clinical exposure to gummy smile cases, including the approximate annual number of patients presenting with a gummy smile complaint, the gender distribution of such patients, and whether they had ever diagnosed a gummy smile incidentally in a patient attending for other dental or periodontal treatments. The final two items addressed professional education and self‐assessment: whether participants considered their undergraduate training on gummy smile diagnosis and management to have been adequate and whether they regarded their current knowledge and clinical skills in this area as sufficient.

The questionnaire for laypeople was shorter in scope. The first section comprised three items covering demographic information: gender, age, and educational background.

The second section comprised the evaluation of 13 photographs, one reference (master) photograph, and 12 modified smile images created through digital alterations of the reference image.

Smile esthetics were assessed using a 10‐cm horizontal visual analog scale (VAS) labeled from 0 (not esthetic) to 10 (esthetic). Participants were asked to rate the esthetic perception of each photograph by marking the appropriate point on a scale. The recorded ratings were expressed on a 0–10 scale and used for statistical analysis.

In addition to the VAS assessment, dental professionals were asked whether each image presented a gummy smile (*Would you diagnose this patient as having a gummy smile?*) and, if so, to indicate the perceived etiology (*In your opinion*, *what is the most likely etiology of the gummy smile in this image?*) with the following categories: skeletal, dental, and soft tissue‐related. Because the assessment was based exclusively on standardized photographs without clinical examination, dynamic smile assessment, periodontal measurements, or radiographic evaluation, participants’ responses were interpreted as image‐based diagnostic impressions rather than definitive clinical diagnoses. Likewise, the reported etiologies should be regarded as participants’ perceived etiologies or etiologic attributions rather than the actual anatomical causes of the gummy smile.

The questionnaire included skeletal, dental, and soft tissue‐related categories as response options for the perceived etiology of the gummy smile. No formal definitions or explanatory information regarding these categories was provided to the participants. The response options were presented as listed in the questionnaire, and participants were asked to select the category that best reflected their own perception of the underlying cause.

The study aimed to evaluate individual esthetic perception rather than measurement reproducibility. Therefore, each participant evaluated the images only once to capture their spontaneous perception of smile esthetics.

This study was reported in accordance with the STROBE guidelines (see Table S2).

### 2.5. Statistical Analysis

Statistical analyses were performed using MedCalc Statistical Software version 22.009 (MedCalc Software Ltd., Ostend, Belgium) and SPSS (version 31.0; IBM Corp., Armonk, NY, USA). The Shapiro–Wilk test was used to assess the normality of continuous data. Since VAS scores did not follow a normal distribution, nonparametric tests were applied throughout the analyses.

Descriptive statistics for VAS esthetic scores are presented as the median with 25th–75th percentile interquartile ranges (IQRs). Categorical variables are expressed as frequencies and percentages.

Differences in VAS esthetic scores among the five study groups (laypeople, GP dentists, periodontists, orthodontists, and prosthodontists) were evaluated using the Kruskal–Wallis *H* test. The effect size was calculated as *η* ^2^ = *H*/(*N* − 1), where values of <0.06, 0.06–0.14, and >0.14 indicate small, moderate, and large effects, respectively. When significant differences were detected, post hoc pairwise comparisons were performed using the Mann–Whitney *U* test with Bonferroni correction. Given the multiple comparisons performed across photographs and participant groups, post hoc pairwise comparisons were adjusted using the Bonferroni correction to reduce the risk of type I error. In addition, each photograph was analyzed independently according to the predefined objectives of the study as the primary aim was to compare participants’ responses for each image rather than to evaluate within‐subject changes across photographs.

To compare VAS scores between laypeople and dental professionals as a combined group, Mann–Whitney *U* tests were performed, and effect size was calculated as *r* = *Z*/√*N*, with values of <0.1, 0.1–0.3, 0.3–0.5, and >0.5 interpreted as negligible, small, moderate, and large, respectively.

Intragroup comparisons of VAS scores according to gender were conducted using the Mann–Whitney *U* test, while age and professional experience subgroup analyses were performed using the Kruskal–Wallis test. Pairwise percentile comparisons are reported using Tukey’s Hinges method.

Differences in the frequency of the diagnostic impression of a gummy smile (yes/no) across dental professional groups were analyzed using the Chi‐square test. When more than 20% of cells had expected frequencies below 5, the Fisher–Freeman–Halton (FFH) exact test was applied. Photographs in which all professionals unanimously responded (100% yes) were excluded from statistical testing. Photographs in which the total number of diagnostic impressions of gummy smiles was fewer than five across all groups were also excluded from analysis.

Differences in the perceived etiology of gummy smile (skeletal, dental, or soft tissue‐related) among dental professional groups were assessed using the chi‐square test or FFH where applicable, restricted to participants who diagnosed gummy smile in each photograph.

Perceived etiology analysis was restricted to participants who identified an image as representing a gummy smile. For images with sparse data, intergroup statistical comparisons were not performed because the number of observations within one or more professional groups and/or etiologic categories was insufficient to provide reliable and meaningful statistical results.

Knowledge and attitude data were analyzed using the chi‐square test. When significant differences were identified, post hoc pairwise comparisons with Bonferroni correction were applied. Statistical significance was defined as *p*  < 0.05 for all analyses.

## 3. Results

### 3.1. Demographic Characteristics

A total of 250 participants were enrolled in this study, comprising 50 laypeople and 200 dental professionals (50 GP dentists, 50 periodontists, 50 orthodontists, and 50 prosthodontists). Demographic characteristics are presented in Table 1. The overall female‐to‐male ratio was ~3:2 across all groups, and no statistically significant differences in gender distribution were found between groups (*p* = 0.904). The age distribution differed between groups, with laypeople having a higher proportion of participants aged ≥40 years (44%) compared to dental professionals, although this difference did not reach statistical significance across all groups (*p* = 0.436) or among dental professionals alone (*p* = 0.696). With respect to professional experience, the distribution of years in dental practice did not differ significantly among the four dental professional groups (*p* = 0.165).

**Table 1:** Demographic data of individuals.

Demographic characteristics	Laypeople *n* (%)	GP dentists *n* (%)	Periodontists *n* (%)	Orthodontists *n* (%)	Prosthodontists *n* (%)	*p*
Gender						
Female	32 (64)	29 (58)	31 (62)	32 (64)	29 (58)	0.904^c^
Male	18 (36)	21 (42)	19 (38)	18 (36)	21 (42)	
Age						
20–25	9 (18)	3 (6)	3 (6)	—	—	0.436^a^ 0.696^b^
26–32	12 (24)	21 (42)	22 (44)	25 (50)	21 (42)	
33–39	7 (14)	16 (32)	19 (38)	22 (44)	22 (44)	
≥ 40 years	22 (44)	10 (20)	6 (12)	3 (6)	7 (14)	
Experience in dental practice						
1–5 years	—	11 (22)	21 (42)	17 (34)	17 (34)	0.165^b^
6–10 years	—	16 (32)	18 (36)	23 (46)	19 (38)	
11–15 years	—	13 (26)	8 (16)	7 (14)	9 (18)	
≥ 16 years	—	10 (20)	3 (6)	3 (6)	5 (10)	

^a^Kruskal–Wallis (between all groups).

^b^Kruskal–Wallis (between dental professionals).

^c^Chi‐square test (between all groups and only dental professionals).

### 3.2. VAS Esthetic Scores

VAS esthetic scores for all photographs across groups are presented in Table 2. The Kruskal–Wallis test revealed statistically significant differences among all five groups for 12 of the 13 photographs (all *p*  < 0.05), with the exception of photograph #7 (*H* = 5.932, *p* = 0.204). Effect sizes ranged from small (*η* ^2^ = 0.024 for photograph #7) to large (*η* ^2^ = 0.227 for photograph #3). The largest group differences were observed for photographs #1, #2, and #3, which represent progressive increments in gingival display, with large effect sizes (*η* ^2^ = 0.203, 0.215, and 0.227, respectively).

**Table 2:** Descriptive statistics of VAS esthetic scores between groups (median 25th–75th percentile).

Photo	Laypeople	Dental professionals				All groups			Laypeople vs. dental professionals	
		GP dentists	Periodontists	Orthodontists	Prosthodontists	*H*	*p* ^a^	*η* ^2^	*p* ^b^	*r*
Master	8 (8–10)	9 (7–10)	9 (8–10)	7 (6.75–8)	8 (7–9)	24.093	**<0.001** ^∗^	0.097	0.189	0.083
#1	8 (7–9)	7 (6–8)	7 (6–8)	6 (5–7)	7 (6–7.25)	50.658	**<0.001** ^∗^	0.203	**<0.001** ^∗∗^	0.330
#2	7 (5–8)	6 (4–6)	5 (5–6)	4 (3–5)	5 (4–6)	53.451	**<0.001** ^∗^	0.215	**<0.001** ^∗∗^	0.385
#3	5 (4–6)	4 (3–5)	4 (3–5)	3 (2–4)	4 (3–5)	56.604	<0.001^∗^	0.227	**<0.001** ^∗∗^	0.413
#4	4 (2–5)	1.5 (1–3)	2 (1–3)	1.5 (0.75–3)	2 (1–3)	38.402	<0.001^∗^	0.154	**<0.001** ^∗∗^	0.341
#5	8 (7–8)	8 (7–9)	8 (7–8)	8 (7–8)	8 (8–9)	13.742	0.008^∗^	0.055	0.096	0.105
#6	8 (7–9)	8 (7–8)	7 (6–8)	7 (6–9)	7 (7–8)	9.551	0.049^∗^	0.038	**0.020** ^∗∗^	0.147
#7	8 (7–9)	8 (7.75–9)	8 (8–9)	8 (7–8)	8 (7–9)	5.932	0.204	0.024	0.281	0.068
#8	8 (7–9)	8 (7–9)	9 (8–10)	8 (7–9)	9 (8–9)	13.331	0.010^∗^	0.054	**0.022** ^∗∗^	0.145
#9	7 (6.75–8)	7.5 (7–8)	8 (7–10)	8 (7–9)	8 (7–9)	19.613	<0.001^∗^	0.079	**<0.001** ^∗∗^	0.223
#10	6 (5–8)	6 (5–7)	7 (6–7)	6 (5–7)	5 (4–5)	16.237	0.003^∗^	0.065	0.480	0.045
#11	5 (3.75–6)	4 (3–5)	5 (4–6)	4 (4–5)	5 (4–5)	13.575	0.009^∗^	0.055	0.280	0.068
#12	3 (1–5)	1 (1–3)	3 (2–4)	2 (1–3)	3 (2–3)	23.447	**<0.001** ^∗^	0.094	0.100	0.104

*Note:* Data are presented as median (25th–75th percentiles). Bold values indicate statistically significant differences.

^a^Kruskal–Wallis test (between all five groups).

^b^Mann–Whitney *U* test (laypeople vs. all dental professionals combined).

^∗^ *p*  < 0.05. Effect size *η* ^2^ *= H/*(*N* − 1): < 0.06 small, 0.06–0.14 moderate, > 0.14 large.

^∗∗^ *p* < 0.05. Effect size *r* = *Z*/√*N*: < 0.1 negligible, 0.1–0.3 small, 0.3–0.5 moderate, > 0.5 large.

When comparing laypeople to dental professionals as a single group, significant differences were observed for photographs #1–4 (all *p*  < 0.001), #6 (*p* = 0.020), #8 (*p* = 0.022), and #9 (*p*  < 0.001). Laypeople consistently assigned higher esthetic scores to photographs with increased gingival display (photographs #1–4) compared to dental professionals, with moderate effect sizes (*r* = 0.330–0.413 for photographs #1–4). Conversely, for photographs representing increased gingival display in the upper lip region (#8 and #9), dental professionals rated the esthetic appearance higher than laypeople, indicating a different perception of smile attractiveness in relation to upper lip position.

Post hoc pairwise comparisons (Table 3) revealed that orthodontists assigned significantly lower VAS scores than all other groups for photographs #1–4 (all *p*  < 0.05), indicating a more critical esthetic perception of increased gingival exposure. Periodontists also gave significantly lower scores compared to laypeople for several photographs (#1, #2, #3, #4, and #6) and assigned higher scores than orthodontists for photographs representing the master and low gingival display images (Master, #1, #2, and #3). No significant differences were observed between periodontists and prosthodontists in any photograph. Prosthodontists rated photographs #1–4 significantly lower than laypeople (all *p* ≤ 0.012), while GP dentists differed from laypeople only for photographs #2, #3, #4, and #12.

**Table 3:** Post hoc pairwise comparisons between groups (Mann–Whitney *U* test with Bonferroni correction).

Comparison	Photographs												
	Master	#1	#2	#3	#4	#5	#6	#7	#8	#9	#10	#11	#12
Laypeople vs. GP dentists	1.00	0.084	0.002^∗^	<0.001^∗^	<0.001^∗^	0.522	1.00	1.00	1.00	1.00	1.00	0.242	0.004^∗^
Periodontists vs. GP dentists	1.00	1.00	1.00	1.00	0.250	1.00	0.431	1.00	1.00	0.076	0.087	0.009^∗^	<0.001^∗^
Periodontists vs. orthodontists	<0.001^∗^	<0.001^∗^	0.009^∗^	0.010^∗^	0.274	1.00	1.00	0.104	1.00	1.00	0.005^∗^	0.083	0.076
Periodontists vs. prosthodontists	1.00	1.00	1.00	1.00	1.00	0.403	1.00	1.00	1.00	1.00	1.00	0.857	1.00
Periodontists vs. laypeople	1.00	0.031^∗^	0.001^∗^	<0.001^∗^	0.018^∗^	1.00	0.035^∗^	0.040^∗^	1.00	<0.001^∗^	0.242	1.00	1.00
Orthodontists vs. GP dentists	0.001^∗^	<0.001^∗^	0.004^∗^	0.129	1.00	0.268	1.00	1.00	1.00	1.00	1.00	1.00	1.00
Orthodontists vs. prosthodontists	0.041^∗^	<0.001^∗^	0.030^∗^	0.020^∗^	0.371	0.020^∗^	1.00	0.246	1.00	1.00	0.056	1.00	0.521
Orthodontists vs. laypeople	0.002^∗^	<0.001^∗^	<0.001^∗^	<0.001^∗^	<0.001^∗^	1.00	0.848	1.00	1.00	0.048^∗^	1.00	1.00	0.358
Prosthodontists vs. GP dentists	1.00	1.00	1.00	1.00	0.339	1.00	1.00	1.00	1.00	1.00	0.539	1.00	0.008^∗^
Prosthodontists vs. laypeople	1.00	0.001^∗^	<0.001^∗^	<0.001^∗^	0.012^∗^	0.047^∗^	0.707	0.104	1.00	0.049^∗^	1.00	1.00	1.00

*Note:* Bold values indicate statistically significant differences.

^∗^ *p* < 0.05.

### 3.3. Diagnostic Impression of Gummy Smile (Perceived Gummy Smile)

The frequency of the perceived gummy smile is presented in Table 4 and varied across photographs, with unanimous agreement observed only for photographs #3, #4, and #12, indicating a clear diagnostic threshold at higher levels of gingival display.

**Table 4:** Analysis of responses to the question, “Would you diagnose this patient as having a gummy smile?” by dental professional groups.

Photographs	GP dentists *n* (%)	Periodontists *n* (%)	Orthodontists *n* (%)	Prosthodontists *n* (%)	Test	*p* ^∗^
Master photo	8 (16)	5 (10)	10 (20)	6 (12)	Chi‐square	0.497
Photo 1	19 (38)	19 (38)	36 (72)	25 (50)	Chi‐square	0.001^∗^
Photo 2	42 (84)	49 (98)	48 (96)	48 (96)	FFH	0.220
Photo 3	50 (100)	50 (100)	49 (98)	50 (100)	FFH	1.000
Photo 4	50 (100)	50 (100)	50 (100)	50 (100)	—^a^	—
Photo 5	8 (16)	—	2 (4)	—	FFH	<0.001
Photo 6	2 (4)	—	—	—	—^b^	—
Photo 7	5 (10)	3 (6)	5 (10)	4 (8)	FFH	0.943
Photo 8	4 (8)	3 (6)	6 (12)	3 (6)	FFH	0.766
Photo 9	2 (4)	1 (2)	—	3 (6)	—^b^	—
Photo 10	—	1 (2)	—	—	—^b^	—
Photo 11	47 (94)	50 (100)	48 (96)	50 (100)	FFH	0.171
Photo 12	50 (100)	50 (100)	50 (100)	50 (100)	—^a^	—

*Note:* FFH: Fisher–Freeman–Halton exact test applied when >20% of cells had expected frequencies <5.

^a^All professionals unanimously diagnosed gummy smile; statistical testing not applicable.

^b^Statistical analysis not performed due to insufficient frequency of gummy smile diagnosis (*n* < 5 across groups).

^∗^ *p*  < 0.05.

For photographs with limited gingival display (Master and #7 and #8), diagnostic impression rates were low across all groups (range: 6%–20%). Photograph #3 demonstrated near‐unanimous agreement, with 98%–100% of participants across groups identifying it as representing a gummy smile (*p* = 1.000). Photographs #4 and #12 were unanimously identified as representing a gummy smile by all professionals (100%), and statistical testing was therefore not applicable. Photographs #6, #9, and #10 had fewer than five perceived gummy smiles across groups and were also excluded from analysis.

Significant intergroup differences in diagnosis frequency were observed for photograph #1 (chi‐square, *p* = 0.001) and photograph #5 (FFH, *p*  < 0.001), with orthodontists demonstrating the highest diagnosis rate for photograph #1 (72%) compared to GP dentists and periodontists (38% each). For photographs #2 and #3, diagnosis rates were uniformly high across all groups (>84%), and no significant differences were observed (*p* = 0.220 and *p* = 1.000, respectively). Photographs #4 and #12 were unanimously judged as having a gummy smile by all professionals (100%). Photograph #3 had near‐unanimous agreement (98%–100% across groups; *p* = 1.000).

### 3.4. Gummy Smile Etiology Perception

Etiology perception among professionals who diagnosed gummy smiles is presented in Table 5. Significant intergroup differences in perceived etiology were identified for the master photograph (FFH, *p* = 0.006), photographs #1 (FFH, *p*  < 0.001), #2 (chi‐square, *p*  < 0.001), #3 (chi‐square, *p*  < 0.001), #4 (chi‐square, *p*  < 0.001), #11 (FFH, *p*  < 0.001), and #12 (FFH, *p*  < 0.001). For photographs #5, #6, #8, #9, and #10, etiology analysis was not performed due to fewer than five gummy smile diagnoses across all professional groups combined. Photograph #5 was excluded from etiology analysis due to insufficient responses within categories, limiting meaningful intergroup comparison (*n* < 5 per group).

**Table 5:** Analysis of responses to the question “In your opinion, what is the most likely etiology of the gummy smile in this image?” by dental professional groups.

Photographs	*n*	GP dentists *n* (%)	Periodontists *n* (%)	Orthodontists *n* (%)	Prosthodontists *n* (%)	Test	*p* ^∗^
Master photo	**29**					FFH	**0.006** ^∗^
Skeletal				2 (20)	2 (33.3)		
Dental				4 (40)	3 (50)		
Soft tissue‐related		8 (100)	5 (100)	4 (40)	1 (16.7)		
Photo 1	**99**					FFH	**<0.001** ^∗^
Skeletal		1 (5.3)		3 (8.3)	5 (20)		
Dental		7 (36.8)	2 (10.5)	5 (13.9)	14 (56)		
Soft tissue‐related		11 (57.9)	17 (89.5)	28 (77.8)	6 (24)		
Photo 2	**187**					Chi‐square	**<0.001** ^∗^
Skeletal		8 (22.9)	2 (5.7)	18 (51.4)	7 (20)		
Dental		12 (29.3)	6 (14.6)	6 (14.6)	17 (41.5)		
Soft tissue‐related		22 (52.4)	41 (37.3)	23 (20.9)	24 (21.8)		
Photo 3	**199**					Chi‐square	**<0.001** ^∗^
Skeletal		11 (22)	7 (14)	27 (54)	9 (18)		
Dental		12 (24)	4 (8)	7 (14)	12 (24)		
Soft tissue‐related		27 (54)	39 (78)	16 (32)	29 (58)		
Photo 4	**200**					Chi‐square	**<0.001** ^∗^
Skeletal		12 (24)	12 (24)	33 (66)	20 (40)		
Dental		10 (20)	2 (4)	3 (6)	8 (16)		
Soft tissue‐related		28 (56)	36 (72)	14 (28)	22 (44)		
Photo 5	**10**					^a^	
Skeletal		3 (33.3)					
Dental		1 (11.1)		2 (100)			
Soft tissue‐related		5 (55.6)					
Photo 6	**2**					^a^	
Skeletal							
Dental							
Soft tissue‐related		2 (100)					
Photo 7	**17**					FFH	0.566
Skeletal			1 (33.3)				
Dental				1 (20)	1 (25)		
Soft tissue‐related		5 (100)	2 (66.7)	4 (80)	3 (75)		
Photo 8	**16**					^a^	
Skeletal							
Dental		3 (75)	1 (33.3)	1 (16.7)	1 (33.3)		
Soft tissue‐related		1 (25)	2 (66.7)	5 (83.3)	2 (66.7)		
Photo 11	**195**					FFH	**<0.001** ^∗^
Skeletal		23 (48.9)	12 (24)	32 (66.7)	27 (54)		
Dental		3 (6.4)	2 (4)	4 (8.3)	1 (2)		
Soft tissue‐related		21 (44.7)	36 (72)	12 (25)	22 (44)		
Photo 12	**200**					FFH	**<0.001** ^∗^
Skeletal		39 (78)	29 (58)	45 (90)	42 (84)		
Dental							
Soft tissue‐related		11 (22)	21 (42)	5 (10)	8 (16)		

*Note:* FFH: Fisher–Freeman–Halton exact test, applied when >20% of cells had expected frequencies <5. Bold values indicate statistically significant differences.

^a^Statistical analysis not performed because the number of observations within one or more groups and/or etiologic categories was insufficient to permit meaningful intergroup comparison.

^∗^ *p*  < 0.05.

A consistent pattern emerged across multiple photographs: orthodontists most frequently attributed gummy smiles to skeletal etiology (e.g., 51.4% for photograph #2, 54% for photograph #3, 49.1% for photograph #4, and 66.7% for photograph #11), while periodontists predominantly identified soft tissue‐related causes (e.g., 89.5% for photograph #1, 37.3% for photograph #2, 78% for photograph #3, and 72% for photograph #11). Prosthodontists showed a higher tendency toward dental etiology compared to other groups, particularly for photographs #1 (56%) and photograph #2 (41.5%). GP dentists demonstrated mixed etiology perceptions, with a predominance of soft tissue‐related attribution in photographs with a moderate gingival display.

For photographs #3 and #4, all or nearly all professionals diagnosed gummy smiles (99%–100%), yet etiology perceptions differed markedly. In photograph #3, periodontists attributed gummy smiles predominantly to soft tissue‐related causes (78%), while orthodontists most commonly identified skeletal etiology (54%). In photograph #12, skeletal etiology was the most frequently selected cause across all groups (range: 58%–90%), with orthodontists showing the highest proportion (90%).

### 3.5. Knowledge and Attitudes

Responses to the knowledge and attitude questionnaire are presented in Table 6. The majority of dental professionals across all groups reported having encountered gummy smile patients in their practice (GP dentists: 88%, periodontists: 98%, orthodontists: 90%, and prosthodontists: 100%). Most reported seeing 1–10 gummy smile patients per year, with no significant differences among groups (*p* = 0.125).

**Table 6:** Evaluation of the knowledge and attitudes of GP dentists and specialist dentists regarding gummy smile.

Questionnaire item	GP dentists *n* (%)	Periodontists *n* (%)	Orthodontists *n* (%)	Prosthodontists *n* (%)	*p* ^∗^
Have you ever received orthodontic treatment?					
No	36 (72)	37 (74)	29 (58)	39 (78)	0.151
Yes	14 (28)	13 (26)	21 (42)	11 (22)	
Have you ever received treatment for a gummy smile?					
No	48 (96)	49 (98)	46 (92)	50 (100)	0.122
Yes	2 (4)	1 (2)	4 (8)	0 (0)	
Approximately how many patients present to your clinic with a complaint of gummy smile per year?					
None	4 (8)	0 (0)	0 (0)	0 (0)	0.125
1–5	23 (46)	30 (60)	31 (62)	22 (44)	
6–10	19 (38)	15 (30)	12 (24)	21 (42)	
11 and more	4 (8)	5 (10)	7 (14)	7 (14)	
What is the predominant gender of the patients who present with the complaint of gummy smile?					
Mostly women	40 (80)	48 (96)	43 (86)	50 (100)	—
Mostly men	0 (0)	0 (0)	1 (2)	0 (0)	
Similar numbers	10 (20)	2 (4)	6 (12)	0 (0)	
Have you ever diagnosed gummy smile incidentally in a patient who sought treatment for other dental or periodontal conditions?					
No	6 (12)	1 (2)	5 (10)	0 (0)	—
Yes	44 (88)	49 (98)	45 (90)	50 (100)	
Do you consider your undergraduate training on gummy smile diagnosis and management to have been adequate?					
No	44 (88)	37 (74)	27 (54)	33 (66)	0.001^∗^
Yes	6 (12)^a^	13 (26)^a,b^	23 (46)^b^	17 (34)^a,b^	
How do you assess your knowledge and clinical skills regarding gummy smile diagnosis and treatment?					
Yes, I have enough knowledge	3 (6)	22 (44)	11 (22)	20 (40)	—
Yes, but I feel inadequate	42 (84)	28 (56)	37 (74)	30 (60)	
No, I do not have any knowledge about it	5 (10)	0 (0)	2 (4)	0 (0)	

*Note:* Groups without a shared letter differ significantly (*p* < 0.05).

^a,b^Bonferroni‐corrected pairwise comparisons: sharing the same superscript letter do not differ significantly from each other (*p* < 0.05).

^∗^Chi‐square test.

A statistically significant difference was observed in the perceived adequacy of undergraduate training on gummy smiles (*p* = 0.001). Orthodontists reported the highest rate of having received adequate training (46%), while GP dentists reported the lowest (12%). Post hoc pairwise comparisons with Bonferroni correction indicated that GP dentists were significantly less likely to report adequate training compared to orthodontists (*p*  < 0.05).

Regarding self‐assessed clinical competence, the majority of participants across all groups reported feeling that they possessed some knowledge but felt inadequate in its application (GP dentists: 84%, periodontists: 56%, orthodontists: 74%, and prosthodontists: 60%). Only 6% of GP dentists reported having sufficient knowledge and skills for gummy smile management, compared to 44% of periodontists, 22% of orthodontists, and 40% of prosthodontists. Nearly all professionals across groups had encountered incidental gummy smile diagnoses during routine dental visits.

### 3.6. Intragroup Comparisons

Intragroup analyses examining the influence of gender, age, and professional experience on VAS esthetic scores are presented in Tables S3–S7. Within periodontists, VAS scores for photographs #5 and #7 differed significantly by gender (*p* = 0.031 and *p* = 0.016, respectively), with male periodontists assigning higher scores. Significant differences by professional experience were observed for photographs #11 (*p* = 0.008) and #12 (*p* = 0.002), while age‐related differences reached significance for photographs #11 (*p* = 0.016) and #12 (*p* = 0.018).

Among orthodontists, gender significantly influenced scores for photograph #5 (*p* = 0.038). Age‐related differences were observed for photographs #4 (*p* = 0.041), #9 (*p* = 0.017), and the master photograph approached significance (*p* = 0.137). Professional experience significantly influenced scores for photographs #11 (*p* = 0.032) and #12 (*p* = 0.031).

Within GP dentists, professional experience significantly influenced VAS scores for photographs #5 (*p* = 0.020), #6 (*p* = 0.020), #7 (*p* = 0.026), and #12 (*p* = 0.009), with more experienced clinicians generally assigning higher scores. Significant age‐related differences were observed for photographs #5 (*p* = 0.017) and #7 (*p* = 0.024). Among prosthodontists, gender significantly influenced scores for the master photograph (*p* = 0.049), and no significant differences by age or experience were observed for any photograph.

## 4. Discussion

The most pronounced intergroup differences in esthetic perception were observed for photographs #1 through #4, representing mild to moderate gingival display (−2 mm to +2 mm relative to the reference image). These photographs yielded the largest effect sizes in the Kruskal–Wallis analyses (*η* ^2^ = 0.203–0.227). This pattern suggests that esthetic perception does not operate as a binary threshold but rather within a transitional zone, where professional training significantly influences judgments.

The variability in diagnosis and etiology attribution observed across photographs further indicates that the clinical threshold for gummy smile recognition may be reached earlier than consensus on its underlying cause, highlighting the importance of etiology‐based treatment planning discussions among dental professionals.

It should be noted that etiology analysis for photograph #5 was not performed because fewer than five gummy smile diagnoses were recorded across all groups when the photograph was re‐evaluated in the context of etiology, likely reflecting differences in how participants interpreted the etiology question relative to the binary diagnosis question.

The present study evaluated the criteria used by GP dentists and dental specialists in diagnosing gummy smiles and compared their esthetic perception of gingival display with that of laypeople. The findings demonstrated significant differences in the esthetic perception among the study groups. Dental professionals generally evaluated gingival display more critically than laypeople [21, 22], and orthodontists assigned lower esthetic scores than other professional groups across most photographs. Median VAS scores for orthodontists were consistently lower than those of laypeople across photographs #1–#4, with differences ranging from ~1.5–2.5 points (Table 2) [10, 23]. In addition, differences were observed among dental specialties regarding the perceived etiology of gummy smile, with orthodontists more frequently attributing the condition to skeletal factors, whereas periodontists and GP dentists more commonly identified soft tissue‐related causes. This difference may reflect the distinct clinical training of various dental specialties.

Smile esthetics play an essential role in facial attractiveness and social perception. Because the smile is one of the most prominent facial expressions, even small variations in gingival display can influence the perceived attractiveness of a smile. Previous studies have demonstrated that dental and gingival components, including the smile arc, buccal corridors, and gingival display, are important determinants of smile esthetics and facial harmony [6, 7]. In particular, several studies have shown that increased gingival display may negatively affect the perception of smile attractiveness [3, 7].

The results of the present study are consistent with previous research showing that dental professionals and laypeople evaluate smile esthetics differently. Kokich et al. [10] reported that dental professionals are more sensitive to minor deviations in dental and gingival structures compared with laypeople. Similarly, Al Taki et al. [24] demonstrated that orthodontists tend to be more critical when evaluating alterations in smile esthetics than GP dentists and laypeople. These findings align with the present results, in which orthodontists consistently assigned lower esthetic scores to several photographs compared with those of other groups.

One possible explanation for this observation is the diagnostic perspective associated with orthodontic training. In addition, these findings may also be interpreted within the framework of perceptual and cognitive biases. Clinical training may condition specialists to focus on specific anatomical components, leading to selective attention toward features aligned with their expertise. This may partially explain the systematic differences observed in both the esthetic evaluation and etiology attribution across specialties. Orthodontists routinely assess dentofacial relationships, skeletal discrepancies, and smile line characteristics during clinical evaluation. This may increase their sensitivity to variations in gingival display and lead them to apply stricter esthetic criteria when evaluating smile attractiveness. In contrast, laypeople may evaluate smiles more holistically, focusing on the overall appearance rather than subtle gingival variations.

Another important finding of this study was the variation in the perceived etiology of gummy smiles among dental specialties. Appropriate treatment depends on accurately identifying the underlying etiology because a gummy smile can originate from several anatomical or functional factors [25]. Although participants frequently agreed that a photograph represented a gummy smile, substantial differences were observed regarding the perceived underlying cause. This finding suggests that the recognition of excessive gingival display may be more consistent than the interpretation of its underlying etiology. Because no formal definitions of etiologic categories were provided, the observed variability may partly reflect differences in how participants conceptualize skeletal, dental, and soft tissue‐related causes of gummy smiles. Consequently, the differences observed among professional groups may reflect not only variations in clinical judgment but also differences in the interpretation of the etiologic categories themselves.

Orthodontists more frequently attributed gummy smiles to skeletal causes, whereas periodontists and GP dentists more commonly selected soft tissue‐related factors. These differences likely reflect the clinical focus of each specialty. Orthodontists often emphasize skeletal and dentofacial relationships during diagnosis, whereas periodontists primarily evaluate gingival morphology and soft tissue architecture. Similar differences in diagnostic perspectives among dental specialties have been reported in previous studies evaluating smile esthetics [2, 8].

The higher tendency of prosthodontists to attribute gummy smiles to dental causes warrants further consideration. Prosthodontists routinely manage patients with tooth wear, crown lengthening requirements, and altered passive eruption, conditions in which the apparent increase in gingival display is predominantly related to changes in clinical crown length rather than skeletal or soft tissue factors. This clinical background may predispose prosthodontists to interpret gingival display within a restorative framework, emphasizing dental dimension alterations as the primary contributing factor. The present findings suggest that specialty‐specific clinical exposure may systematically bias etiology attribution, which has direct implications for treatment planning. This observation may have implications for treatment planning approaches across specialties, although further investigation is required. This underscores the critical importance of multidisciplinary assessment in gummy smile diagnosis to ensure that treatment planning reflects the true underlying etiology rather than a specialty‐driven perspective.

A particularly noteworthy finding was the unanimous gummy smile diagnosis in photograph #12 by all dental professionals (100%). Photograph #12 was generated by shifting the upper lip position 2 mm inferiorly relative to the master photograph, resulting in greater gingival exposure without any alteration to the underlying hard‐ or soft‐tissue architecture. The universal diagnostic agreement in the diagnostic impression observed for this image is clinically significant, as it implies that dental professionals consistently perceive an inferiorly displaced lip position as sufficient to warrant a gummy smile diagnosis, regardless of their specialty. This observation is consistent with the widely accepted classification of gummy smile etiology proposed by Peck et al. [11], which identifies hypermobility of the upper lip and altered lip length as independent etiological contributors. Interestingly, despite unanimous diagnostic consensus, etiology perceptions across groups differed significantly (FFH, *p*  < 0.001), with skeletal attribution predominating across all specialties. This finding is noteworthy because the photographic modification was exclusively lip‐based and did not involve any skeletal, dental, or gingival alterations. One possible explanation is that clinicians may rely on heuristic reasoning or specialty‐driven diagnostic frameworks when evaluating excessive gingival display. When confronted with a pronounced gummy smile, clinicians may tend to anchor their diagnostic reasoning to etiologies that are more familiar within their clinical training and experience, even when the available visual information does not directly support such interpretations. This phenomenon may be particularly relevant in smile esthetics, where diagnosis often relies on visual perception and subjective interpretation. Although the present study was not designed to investigate cognitive bias directly, the discrepancy between diagnostic consensus and etiology attribution suggests that clinicians’ prior experiences and specialty‐specific perspectives may influence their interpretation of gummy smile etiology. These findings highlight a potential disconnect between diagnostic recognition and etiology attribution in gummy smile assessment and reinforce the need for systematic clinical protocols that distinguish between the various anatomical contributors to excessive gingival display.

Interestingly, no statistically significant differences were observed among the groups for one of the photographs representing moderate gingival exposure. This finding suggests that small variations in gingival display within a certain range may be perceived similarly by both dental professionals and laypeople. Previous studies have also suggested that gingival exposure within a limited range may be considered esthetically acceptable by both clinicians and non‐professional observers.

Within‐group analyses also revealed that gender, age, and professional experience influenced the esthetic perception in some images. These findings suggest that esthetic judgments may be affected not only by professional training but also by individual characteristics and clinical experience. Previous studies evaluating smile esthetics have similarly reported that demographic variables may influence the esthetic perception [15, 26].

From a clinical perspective, the results highlight the importance of considering patient perception when planning esthetic dental treatments. Although clinicians may evaluate smile characteristics using strict professional criteria, patients may perceive the esthetic outcomes differently. Therefore, effective communication between clinicians and patients is essential to ensure that treatment objectives align with patient expectations and esthetic preferences.

The findings of this study also emphasize the importance of multidisciplinary education in the diagnosis and management of gummy smiles. Differences observed among dental specialties regarding diagnostic criteria and perceived etiology suggest that undergraduate and continuing education programs should incorporate a more integrated approach to smile esthetics and dentofacial evaluation.

This study has several strengths. The use of a single reference photograph allowed standardized manipulation of gingival display while maintaining consistency across all images. The inclusion of multiple dental specialties and a laypeople group enabled a comprehensive comparison of esthetic perception across different observer groups. In addition, the use of standardized digitally modified photographs allowed controlled changes in the gingival display, providing a consistent basis for esthetic evaluation.

Our results suggest that when planning treatment for a mild gummy smile, clinicians should explicitly explore patients’ own esthetic thresholds, as laypeople may accept gingival display that professionals consider excessive. Nevertheless, standardized static photographs remain one of the most widely accepted and frequently used methods in smile esthetics research because of their reproducibility and ability to isolate specific smile variables. However, several authors have emphasized that smiling is a dynamic facial expression and that dynamic records may provide diagnostic information that cannot be captured in static photographs [19, 20].

However, several limitations of the present study should be considered when interpreting the findings. One of the limitations of this study is the strict inclusion and exclusion criteria applied to the laypeople group. Individuals with previous orthodontic treatment or dental cosmetic experience were excluded to minimize potential confounding factors that could influence the esthetic perception independently of gingival display. Previous exposure to orthodontic treatment may increase awareness of smile esthetics or shape individual perceptions through interactions with dental professionals. While this approach allowed the recruitment of a relatively homogeneous lay sample and improved internal validity, it may also have introduced a selection bias and limited the representativeness of the study population. Therefore, caution should be exercised when generalizing the present findings to a broader population. Future studies including more heterogeneous lay populations may provide findings with greater external validity.

Second, smile esthetics in the present study were evaluated using standardized static two‐dimensional photographs. This methodology enabled precise control of gingival display and minimized potential confounding factors associated with interindividual anatomical variations, such as differences in lip morphology, tooth shape, gingival architecture, and facial characteristics [20]. However, static images cannot fully reproduce the dynamic nature of smiling in everyday life. Natural smiles involve complex interactions among lip mobility, facial musculature, speech, and emotional expression, all of which may influence the perception of smile attractiveness and gingival display [5]. Therefore, the esthetic judgments obtained from static photographs may not completely reflect real‐life smile perceptions. Future studies incorporating dynamic video analysis and three‐dimensional imaging techniques may provide a more comprehensive and ecologically valid assessment of smile esthetics and gummy smile perception [19]. Nevertheless, standardized static photographs remain one of the most widely accepted and frequently used methods in smile esthetics research because of their reproducibility and ability to isolate specific smile variables.

The assessment of smile attractiveness is inherently subjective and may vary according to the individual preferences, cultural background, and personal experiences of the observers. Finally, the study sample consisted of participants from a specific population, which may limit the generalizability of the findings to other populations or cultural contexts. Future studies including larger and more diverse samples for cross‐cultural comparison and using dynamic evaluation methods such as video recordings or three‐dimensional imaging may provide a more comprehensive understanding of the perception of smile esthetics and gummy smile diagnosis. Despite these limitations, the study provides valuable insight into differences in the esthetic perception of gingival display among dental professionals and laypeople.

In addition, because each participant evaluated multiple photographs, the observations were correlated within individuals rather than being entirely independent. While each photograph was analyzed separately according to the primary objectives of the study, future studies may benefit from mixed‐effects models that account for within‐subject correlations across repeated measurements. Moreover, despite the use of Bonferroni correction for post hoc analyses, the large number of statistical comparisons performed in the present study may still have increased the risk of type I error. Therefore, the findings should be interpreted with appropriate caution.

Furthermore, because the images represented relative modifications of a reference photograph rather than predetermined absolute gingival display values, it was not possible to establish diagnostic thresholds for gummy smile perception in millimeters. Future studies using calibrated images with predefined gingival display measurements may provide clinically relevant threshold values for different observer groups.

In the present study, all modified images were generated from a single master smile photograph. Although this approach allowed us to isolate the effect of gingival display and minimize confounding factors associated with interindividual anatomical variations, it may limit the generalizability of the findings.

## 5. Conclusions

Within the limitations of the present study, the findings of this study suggest that the perception of gingival display and the diagnosis of gummy smile differ between dental professionals and laypeople, as well as among dental specialties. Orthodontists appear to apply stricter esthetic criteria and more frequently attribute gummy smiles to skeletal causes, whereas periodontists and GP dentists more commonly identify soft tissue‐related factors. These differences highlight the importance of interdisciplinary collaboration and patient‐centered evaluation in the diagnosis and management of gummy smiles. Integrating multidisciplinary education into undergraduate and continuing dental training may improve clinicians’ ability to assess and manage smile esthetics.

## Author Contributions

Conceptualization: Esra Guzeldemir‐Akcakanat. Data curation: Esra Guzeldemir‐Akcakanat and Nimet Turan. Formal analysis: Esra Guzeldemir‐Akcakanat and Nimet Turan. Funding acquisition: Esra Guzeldemir‐Akcakanat and Nimet Turan. Investigation: Nimet Turan. Methodology: Esra Guzeldemir‐Akcakanat and Nimet Turan. Project administration: Esra Guzeldemir‐Akcakanat and Nimet Turan. Resources: Esra Guzeldemir‐Akcakanat and Nimet Turan. Software: Nimet Turan. Supervision: Esra Guzeldemir‐Akcakanat. Validation: Esra Guzeldemir‐Akcakanat and Nimet Turan. Visualization: Esra Guzeldemir‐Akcakanat and Nimet Turan. Writing – original draft: Esra Guzeldemir‐Akcakanat, Priti Charde, and Nimet Turan. Writing – review and editing: Esra Guzeldemir‐Akcakanat.

## Funding

This research received no external funding. Open access funding kindly provided by Qatar University, P.O.Box 2713, Doha, Qatar.

## Disclosure

All authors have read and approved the final version of the manuscript and agreed to the published version of the manuscript. Esra Guzeldemir‐Akcakanat had full access to all of the data in this study and takes complete responsibility for the integrity of the data and the accuracy of the data analysis. This manuscript was derived from the MSc thesis entitled “Evaluation of the perspectives of general practitioners and specialist dentists in diagnosing gummy smiles and comparison of gingival aesthetics perception with the public” by Nimet Turan (2024), conducted under the supervision of Esra Guzeldemir‐Akcakanat.

## Ethics Statement

This study was conducted in accordance with the Declaration of Helsinki and approved by the Ethics Committee of the Medical Faculty of Kocaeli University (Approval Number GOKAEK‐2024/08.10; 08.05.2024).

## Consent

The participants provided their written consent to participate in the study.

## Conflicts of Interest

The authors declare no conflicts of interest.

## Supporting Information

Additional supporting information can be found online in the Supporting Information section.

## Supporting information


**Supporting Information** Table S1: Summary of the image modifications applied to the reference photograph and their relative changes in gingival display. Table S2: The STROBE checklist. Table S3: Comparison of GP dentists’ esthetic scores according to gender, age, and professional experience. Table S4: Comparison of periodontists’ esthetic scores according to gender, age, and professional experience. Table S5: Comparison of orthodontists’ esthetic scores according to gender, age, and professional experience. Table S6: Comparison of prosthodontists’ esthetic scores according to gender, age, and professional experience. Table S7: Pairwise comparisons between the study groups

## Data Availability

The authors confirm that the data supporting the findings of this study are available from the corresponding author upon reasonable request.
