# Supplementary material for: Esthetic Perception of Gingival Display by Laypeople and Diagnostic Impressions and Etiologic Attribution of Gummy Smile by Dental Professionals: A Cross‐Sectional Study
Source: Int J Dent. 2026 Aug 4;2026:4499170. doi: 10.1155/ijod/4499170 (PMC13438864; doi:10.1155/ijod/4499170)
Supplement: Supplementary file 1 [file IJOD-2026-4499170-s001.zip › Supplementary Tables.docx]

| **Photo*** | **Altered structure** | **Direction** | **Relative change (mm)** |
| --- | --- | --- | --- |
| #1 | Gingival margin | Apical | −1 |
| #2 | Gingival margin | Apical | −2 |
| #3 | Gingival margin | Coronal | +1 |
| #4 | Gingival margin | Coronal | +2 |
| #5 | Gingival margin | Coronal | +3 |
| #6 | Gingival margin | Coronal | +4 |
| #7 | Upper lip | Coronal | +1 |
| #8 | Upper lip | Coronal | +2 |
| #9 | Upper lip | Coronal | +3 |
| #10 | Upper lip | Coronal | +4 |
| #11 | Upper lip | Apical | -1 |
| #12 | Upper lip | Apical | -2 |

**Supplementary Table 1.** Characteristics of the digitally modified smile photographs, including the altered structure, direction of change, and magnitude of modification relative to the master (reference) photograph.

*****All modifications are expressed relative to the master (reference) photograph. The master image was not assigned an absolute gingival-display value; therefore, the modified images represent relative changes rather than predetermined absolute gingival-display values.

**Supplementary Table 3.** Comparison of GP dentists` aesthetic scores according to gender, age, and professional experience.

| GP Dentists | Gender | | | | Age | | | | | | Experience | | | | |
| --- | --- | --- | --- | --- | --- | --- | --- | --- | --- | --- | --- | --- | --- | --- | --- |
|  | **Female**  **(n = 29 )** | **Male**  **(n = 21 )** | ***P**** | **20-25**  **(n = 3 )** | | **26-32**  **(n =21 )** | **33-39**  **(n = 16)** | **≥40**  **(n =10)** | ***P+*** | **1-5 years**  **(n =11)** | | **6-10 years**  **(n =16 )** | **11-15**  **years**  **(n =13 )** | **≥16 years**  **(n =10)** | ***P+*** |
| Master Photo |  |  | 0.723 |  | |  |  |  | 0.330 |  | |  |  |  | 0.157 |
| Med (25th-75th percentile) | 9 (8­10) | 9 (8­10) |  | 9 (NA) | | 9 (7-10) | 8 (7.5-10) | 9.5 (8-10) |  | 7 (5-9) | | 9 (8­10) | 8 (7-10) | 9.5 (8­10) |  |
| Photo 1 |  |  | 0.083 |  | |  |  |  | 0.064 |  | |  |  |  | 0.076 |
| Med (25th-75th percentile) | 8 (6­9) | 7 (5­9) |  | 5 (NA) | | 7 (6-8) | 7 (6.5-8) | 8 (7-9) |  | 6 (4­7) | | 7 (6.5-8) | 7 (6-8) | 8 (7­9) |  |
| Photo 2 |  |  | 0.215 |  | |  |  |  | 0.092 |  | |  |  |  | 0.094 |
| Med (25th-75th percentile) | 6 (4­8) | 5 (3­7) |  | 4 (NA) | | 5 (4-6) | 6 (4.5-6) | 6 (4.5-6) |  | 4 (3-6) | | 6 (4.5-6) | 6 (4-6) | 6 (5-7) |  |
| Photo 3 |  |  | 0.563 |  | |  |  |  | 0.088 |  | |  |  |  | 0.087 |
| Med (25th-75th percentile) | 4 (3­5) | 4 (2­5) |  | 3 (NA) | | 4 (3-4) | 4 (3-4.5) | 5 (4-5) |  | 4 (2-4) | | 4 (3-4.5) | 3(3-4) | 5 (4-5) |  |
| Photo 4 |  |  | 0.745 |  | |  |  |  | 0.978 |  | |  |  |  | 0.578 |
| Med (25th-75th percentile) | 2 (1­3) | 1 (0­2) |  | 3 (NA) | | 1 (1-2) | 2 (1-2.5) | 1 (1-2) |  | 2 (1-3) | | 1 (1-2) | 2 (1-2) | 1 (1-2) |  |
| Photo 5 |  |  | 0.877 |  | |  |  |  | 0.017 |  | |  |  |  | **0.020*+*** |
| Med (25th-75th percentile) | 8 (7­9) | 8 (7­9) |  | 7 (NA) | | 8 (6-9) | 8 (8-9) | 9 (8-9) |  | 7 (5-8) | | 8 (8-9) | 8 (8-9) | 9 (8-9) |  |
| Photo 6 |  |  | 0.159 |  | |  |  |  | 0.056 |  | |  |  |  | **0.020*+*** |
| Med (25th-75th percentile) | 7(6­9) | 8 (7­9) |  | 7 (NA) | | 7 (7-8) | 8 (7-8) | 9 (8.5-9) |  | 7 (6.5-7) | | 7.5 (7-8.5) | 8.5 (7­8) | 8.5 (8­9) |  |
| Photo 7 |  |  | 0.902 |  | |  |  |  | **0.024*+*** |  | |  |  |  | **0.026*+*** |
| Med (25th-75th percentile) | 8 (7­9) | 8 (7­9) |  | 8 (NA) | | 8 (7-8) | 8.5 (8-9) | 9 (8-9) |  | 8 (6.5-8.5) | | 8 (7.5-9) | 8.5 (8-9) | 9 (8-9) |  |
| Photo 8 |  |  | 0.639 |  | |  |  |  | 0.182 |  | |  |  |  | 0.187 |
| Med (25th-75th percentile) | 8 (7­9) | 8 (7­9) |  | 8 (NA) | | 8 (7-9) | 9 (8-9) | 9 (8-9.25) |  | 8 (6.5-8.5) | | 8 (7.5-9) | 9 (8-9) | 9 (8.5-9.25) |  |
| Photo 9 |  |  | 0.838 |  | |  |  |  | 0.320 |  | |  |  |  | 0.124 |
| Med (25th-75th percentile) | 8 (7­9) | 7 (6­9) |  | 8 (NA) | | 8 (7-9) | 9 (8-9) | 9 (8-9) |  | 7 (6.5-8.5) | | 8 (7.5­9) | 9 (8­9) | 9 (8-9) |  |
| Photo 10 |  |  | 0.888 |  | |  |  |  | 0.811 |  | |  |  |  | 0.108 |
| Med (25th-75th percentile) | 6 (5-7) | 6 (5-7) |  | 9 (NA) | | 6 (5-7.5) | 8 (7-9) | 7.5 (7-8) |  | 8 (6.5­8) | | 7 (7­7.5) | 8 (7-9) | 7.5 (7-8) |  |
| Photo 11 |  |  | 0.927 |  | |  |  |  | 0.226 |  | |  |  |  | 0.088 |
| Med (25th-75th percentile) | 4 (3-5) | 4 (3­6) |  | 9 (NA) | | 6 (5-7) | 6 (5-7.75) | 6 (5-6.25) |  | 7 (5.5-8.5) | | 5 (4-6.5) | 6 (5-8) | 6 (5-6.25) |  |
| Photo 12 |  |  | 0.832 |  | |  |  |  | 0.226 |  | |  |  |  | **0.009***+* |
| Med (25th-75th percentile) | 1 (0­2) | 1 (0­2) |  | 3 (NA) | | 1 (1-3) | 1 (0-3) | 1.5 (1-2.5) |  | 3 (2-3.5) | | 1 (0- 1.75) | 1 (0-2.5) | 1.5 (1-2) |  |

**Mann-Whitney U test for gender comparisons; +Kruskal-Wallis test for age and experience comparisons.*

**Supplementary Table 4.** Comparison of periodontists` aesthetic scores according to gender, age, and professional experience.

| Periodontists | Gender | | | Age | | | | | | Experience | | | | | |
| --- | --- | --- | --- | --- | --- | --- | --- | --- | --- | --- | --- | --- | --- | --- | --- |
|  | **Female**  **(n = 31)** | **Male**  **(n = 19)** | ***p**** | **20 - 25**  **(n = 3)** | **26 - 32 (n = 22)** | **33 - 39 (n = 19)** | **≥ 40**  **(n = 6)** | ***p+*** | **1 - 5**  **years**  **(n = 21)** | | **6 - 10 years**  **(n = 18)** | **11 - 15 years**  **(n = 8)** | **≥ 16 years**  **(n = 3)** | ***P+*** |  |
| Master Photo |  |  | 0.926 |  |  |  |  | 0.687 |  | |  |  |  | 0.942 |  |
| Med  (25th-75th percentile) | 9  (8­10) | 9  (8.5­9.5) |  | 9  (NA) | 8.5  (7-9.25) | 9  (8–9) | 9.5  (7.5–10.00) |  | 9  (8­9) | | 9  (8­10) | 8.5  (8­9) | 10  (NA) |  |  |
| Photo 1 |  |  | 0.933 |  |  |  |  | 0.835 |  | |  |  |  | 0.880 |  |
| Med  (25th-75th percentile) | 7  (6-8) | 7  (6-8) |  | 7  (NA) | 7  (6–8) | 7  (6–7) | 7.5  (6.5–8) |  | 7  (6-8) | | 7  (7-7) | 7  (6.5-7.5) | 7  (NA) |  |  |
| Photo 2 |  |  | 0.848 |  |  |  |  | 0.903 |  | |  |  |  | 0.259 |  |
| Med  (25th-75th percentile) | 5  (4.5-5.5) | 5  (4.5-5.5) |  | 5  (NA) | 5  (4-6) | 5  (5-6) | 5.5  (4.75–6.25) |  | 5  (4-6) | | 5  (5­6) | 6  (5-6) | 5  (NA) |  |  |
| Photo 3 |  |  | 0.942 |  |  |  |  | 0.424 |  | |  |  |  | 0.422 |  |
| Med  (25th-75th percentile) | 4  (3-5) | 4  (3-5) |  | 4  (NA) | 3.5  (3–5) | 4  (4–4) | 4  (3.75–5.25) |  | 4  (4-5) | | 4  (3­4) | 4  (4-5) | 4  (NA) |  |  |
| Photo 4 |  |  | 0.150 |  |  |  |  | 0.117 |  | |  |  |  | 0.258 |  |
| Med  (25th-75th percentile) | 3  (2.5-3.5) | 2  (1-3) |  | 3  (NA) | 2  (1–3) | 2  (1–3) | 3  (2-4.25) |  | 3  (2-3) | | 2  (1­3) | 3  (2.5­4) | 3  (NA) |  |  |
| Photo 5 |  |  | **0.031*** |  |  |  |  | 0.630 |  | |  |  |  | 0.556 |  |
| Med  (25th-75th percentile) | 7  (6.5-7.5) | 8  (7.5-8.5) |  | 8  (NA) | 8  (7-9) | 8  (7-8) | 7.5  (6.5-8) |  | 8  (7-9) | | 8  (7­9) | 8  (7.5­8) | 7  (NA) |  |  |
| Photo 6 |  |  | 0.095 |  |  |  |  | 0.324 |  | |  |  |  | 0.275 |  |
| Med  (25th-75th percentile) | 7  ( 6.5-7.5) | 7  (6.5-7.5) |  | 9  (NA) | 7  (6-8) | 7  (7-7) | 6.5  (6-7.5) |  | 7  (6-8) | | 7  (7-8) | 7  (7-8.5) | 6  (NA) |  |  |
| Photo 7 |  |  | **0.016*** |  |  |  |  | 0.753 |  | |  |  |  | 0.926 |  |
| Med  (25th-75th percentile) | 8  (7.5-8.5) | 9  (8.5-9.5) |  | 8  (NA) | 8  (7.75-9) | 8  (7-9) | 8  (8-9) |  | 8  (8-9) | | 8  (7.5-9) | 8  (7.25-8.75) | 6.5  (NA) |  |  |
| Photo 8 |  |  | 0.062 |  |  |  |  | 0.660 |  | |  |  |  | 0.634 |  |
| Med  (25th-75th percentile) | 8  (7.5-8.5) | 9  (8-10) |  | 10  (NA) | 8.5  (7.75-9) | 8  (8-9) | 9.5  (7-10) |  | 8  (8-9) | | 9  (8-9) | 8.5  (6.5-8.5) | 8  (NA) |  |  |
| Photo 9 |  |  | 0.074 |  |  |  |  | 0.894 |  | |  |  |  | 0.069 |  |
| Med  (25th-75th percentile) | 8  (7-9) | 9  (8­10) |  | 9  (NA) | 8.5  (7-10) | 8  (7-10) | 8  (7.5-9) |  | 9  (8-9) | | 8  (8-9) | 9  (8.5­10) | 8  (NA) |  |  |
| Photo 10 |  |  | 0.667 |  |  |  |  | 0.251 |  | |  |  |  | 0.782 |  |
| Med  (25th-75th percentile) | 7  (6.5-7.5) | 7  (6-8) |  | 8  (NA) | 7  (6-7.25) | 7  (6-7) | 7  (6.75-7.25) |  | 9  (7.5-10) | | 8  (7-9) | 8.5  (8-9.5) | 8  (NA) |  |  |
| Photo 11 |  |  | 0.582 |  |  |  |  | **0.016*+*** |  | |  |  |  | **0.008*+*** |  |
| Med  (25th-75th percentile) | 5  (4-6) | 5  (4-6) |  | 8  (NA) | 5  (5-7) | 5  (3-5) | 5  (4.75-5.25) |  | 6  (5-7) | | 4.5  (4-5) | 5  (4.5-5.5) | 5  (NA) |  |  |
| Photo 12 |  |  | 0.086 |  |  |  |  | **0.018*+*** |  | |  |  |  | **0.002*+*** |  |
| Med  (25th-75th percentile) | 4  (3.5-4.5) | 2  (1-3) |  | 6  (NA) | 4  (2-5) | 2  (1-4) | 3.5  (2.75-4.00) |  | 4  (3­5.5) | | 2  (1-3) | 3  (1-5) | 3  (NA) |  |  |

****Mann-Whitney U test for gender comparisons; +Kruskal-Wallis test for age and experience comparisons.* NA: Not applicable, due to very small sample size.

**Supplementary Table 5.** Comparison of orthodontists` aesthetic scores according to gender, age, and professional experience.

| Orthodontists | Gender | | | Age | | | | Experience | | | | |
| --- | --- | --- | --- | --- | --- | --- | --- | --- | --- | --- | --- | --- |
|  | **Female**  **(n = 32 )** | **Male**  **(n = 18 )** | ***P**** | **26 - 32**  **(n = 25 )** | **33 - 39**  **(n = 22 )** | **≥ 40**  **(n = 3 )** | ***P+*** | **1 - 5**  **years**  **(n = 17 )** | **6 - 10 years**  **(n=23 )** | **11 - 15 years**  **(n = 7)** | **≥ 16 years**  **(n = 3)** | ***P+*** |
| Master Photo |  |  | 0.112 |  |  |  | 0.137 |  |  |  |  | 0.282 |
| Med  (25th-75th percentile) | 7.5 (6.5-8.5) | 7 (6­8) |  | 7 (5-8) | 8 (7-9) | 8 (NA) |  | 7 (5-7) | 8 (7-8) | 7 (6-8.5) | 8 (NA) |  |
| Photo 1 |  |  | 0.123 |  |  |  | 0.185 |  |  |  |  | 0.919 |
| Med  (25th-75th percentile) | 6 (5-7) | 5 (4.38-5.63) |  | 5 (4-6) | 6 (5-7) | 6 (NA) |  | 6 (5-6) | 6 (5­6.5) | 6 (5­7) | 6 (NA) |  |
| Photo 2 |  |  | 0.172 |  |  |  | 0.604 |  |  |  |  | 0.722 |
| Med  (25th-75th percentile) | 4 (3.5-4.5) | 4 (3-5) |  | 4 (3-5) | 4 (4-5) | 3 (NA) |  | 5 (4-5) | 4 (3.5-5) | 5 (4-5) | 5 (NA) |  |
| Photo 3 |  |  | 0.582 |  |  |  | 0.249 |  |  |  |  | 0.654 |
| Med  (25th-75th percentile) | 3 (2-4) | 3 (2-4) |  | 3 (2-4) | 2.5 (2-3) | 2 (NA) |  | 3 (2-4) | 3 (2-3.5) | 2 (2-3) | 3 (NA) |  |
| Photo 4 |  |  | 0.901 |  |  |  | **0.041** |  |  |  |  | 0.140 |
| Med  (25th-75th percentile) | 1 (0­2) | 2 (0.5­3.5) |  | 2 (1-3) | 1 (0-2) | 1 (NA) |  | 2 (1-3) | 1 (0­2) | 1 (1­2.5) | 3 (NA) |  |
| Photo 5 |  |  | **0.038*** |  |  |  | 0.389 |  |  |  |  | 0.235 |
| Med  (25th-75th percentile) | 8 (7.5-8.5) | 7 (5.88-8.13) |  | 8 (7-8) | 8 (7-8) | 7 (NA) |  | 8 (7-8) | 8 (7.5-8) | 7 (6­7.5) | 7 (NA) |  |
| Photo 6 |  |  | 0.127 |  |  |  | 0.149 |  |  |  |  | 0.710 |
| Med  (25th-75th percentile) | 7 (5.63-8.38) | 8 (7-9) |  | 7 (6-9) | 7.5 (7-9) | 6 (NA) |  | 8 (7-8) | 8 (6.5­8) | 7 (6­7.5) | 7 (NA) |  |
| Photo 7 |  |  | 0.735 |  |  |  | 0.092 |  |  |  |  | 0.618 |
| Med  (25th-75th percentile) | 8 (7.5­8.5) | 7.5 (6.5-8.5) |  | 7 (7-8) | 8 (8-9) | 8 (NA) |  | 7 (6-9) | 8 (6.5-8) | 7 (6.5-7.5) | 6 (NA) |  |
| Photo 8 |  |  | 0.836 |  |  |  | 0.068 |  |  |  |  | 0.413 |
| Med  (25th-75th percentile) | 8 (7-9) | 8 (7-9) |  | 7 (7-8) | 8 (8-9) | 8 (NA) |  | 8 (7-8) | 8 (7.5-9) | 8 (7.5­9) | 8 (NA) |  |
| Photo 9 |  |  | 0.375 |  |  |  | **0.017** |  |  |  |  | 0.372 |
| Med  (25th-75th percentile) | 8 (7-9) | 8.5 (7.75-9.25) |  | 7 (6-8) | 8 (8-9) | 10 (NA) |  | 8 (7-8) | 8 (7-8.5) | 9 (8­9) | 8 (NA) |  |
| Photo 10 |  |  | 0.303 |  |  |  | 0.483 |  |  |  |  | 0.240 |
| Med  (25th-75th percentile) | 6 (5­7) | 6 (5-7) |  | 6 (5-6) | 6 (5-7) | 7 (NA) |  | 8 (7.5-8) | 8 (7-9.5) | 9 (8-9.5) | 8 (NA) |  |
| Photo 11 |  |  | 0.448 |  |  |  | 0.212 |  |  |  |  | **0.032*+*** |
| Med  (25th-75th percentile) | 4 (3.5­4.5) | 4 (3.38-4.63) |  | 4 (4-5) | 4 (3-5) | 6 (NA) |  | 4 (4-6) | 6 (5-7) | 4 (3.5­5) | 6 (NA) |  |
| Photo 12 |  |  | 0.143 |  |  |  | 0.078 |  |  |  |  | **0.031*+*** |
| Med  (25th-75th percentile) | 2 (0.5-3.5) | 2 (1.38-2.63) |  | 3 (2-3.5) | 1 (1-2) | 4 (NA) |  | 2 (1-3) | 2 (1-3) | 1 (0­2.5) | 4 (NA) |  |

**Mann-Whitney U test for gender comparisons; +Kruskal-Wallis test for age and experience comparisons.*

NA = Not applicable, due to very small sample size

**Supplementary Table 6.** Comparison of prosthodontists` aesthetic scores according to gender, age, and professional experience.

| Prosthodontists | Gender | | | Age | | | | | Experience | | | | |
| --- | --- | --- | --- | --- | --- | --- | --- | --- | --- | --- | --- | --- | --- |
|  | **Female**  **(n = 29 )** | **Male**  **(n = 21 )** | ***P**** | **26-32**  **(n = 21 )** | **33-39**  **(n = 22 )** | **≥ 40**  **(n = 7 )** | ***P+*** | **1-5**  **years**  **(n = 17 )** | | **6-10 years**  **(n = 19)** | **11-15 years**  **(n = 9)** | **≥ 16 years**  **(n = 5)** | ***P+*** |
| Master Photo |  |  | **0.049*** |  |  |  | 0.805 |  | |  |  |  | 0.338 |
| Med (25th-75th percentile) | 8 (7-9) | 8 (7-9) |  | 8 (7-9) | 8 (7-9) | 8 (NA) |  | 9 (8­10) | | 8 (7-9) | 8 (8-8) | 8 (NA) |  |
| Photo 1 |  |  | 0.157 |  |  |  | 0.723 |  | |  |  |  | 0.342 |
| Med(25th-75th percentile) | 7 (6.25-7.75) | 6 (4.5-7.5) |  | 7 (6-8) | 7 (6-7) | 7 (NA) |  | 7 (6-8) | | 7 (5.5-7) | 7 (6-7) | 7 (NA) |  |
| Photo 2 |  |  | 0.547 |  |  |  | 0.326 |  | |  |  |  | 0.608 |
| Med(25th-75th percentile) | 5 (4.5-5.5) | 5 (4-6) |  | 5 (4-6) | 5 (4-6) | 6 (NA) |  | 6 (5-6) | | 5 (4-5.5) | 6 (4-6) | 6 (NA) |  |
| Photo 3 |  |  | 0.405 |  |  |  | 0.205 |  | |  |  |  | 0.547 |
| Med (25th-75th percentile) | 4 (3-5) | 3 (2-4) |  | 4 (3-5) | 4 (3-5) | 5 (NA) |  | 4 (3-5) | | 4 (3-4) | 4 (4-5) | 5 (NA) |  |
| Photo 4 |  |  | 0.450 |  |  |  | 0.124 |  | |  |  |  | 0.415 |
| Med (25th-75th percentile) | 2 (1-3) | 2 (0.75­3.25) |  | 2 (1-3) | 2 (2-3) | 3 (NA) |  | 2 (1-3) | | 2 (2-3) | 3 (2-4) | 3 (NA) |  |
| Photo 5 |  |  | 0.893 |  |  |  | 0.973 |  | |  |  |  | 0.527 |
| Med (25th-75th percentile) | 8 (7.5­8.5) | 8 (7.25-8.75) |  | 8 (8-9) | 8 (8-9) | 8 (NA) |  | 9 (8-9) | | 8 (8-9) | 8 (8-9) | 8 (NA) |  |
| Photo 6 |  |  | 0.561 |  |  |  | 0.254 |  | |  |  |  | 0.299 |
| Med (25th-75th percentile) | 7 (6-8) | 8 (7.5-8.5) |  | 7 (6-8) | 7.5 (7-9) | 8 (NA) |  | 8 (7-8) | | 7 (7-8) | 8 (7­8) | 8 (NA) |  |
| Photo 7 |  |  | 0.324 |  |  |  | 0.818 |  | |  |  |  | 0.436 |
| Med (25th-75th percentile) | 8 (7.5-8.5) | 8 (7.25-8.75) |  | 8 (7-9) | 8 (8-9) | 8 (NA) |  | 8 (6-8) | | 7 (7-8) | 8 (7-8) | 8 (NA) |  |
| Photo 8 |  |  | 0.822 |  |  |  | 0.631 |  | |  |  |  | 0.373 |
| Med (25th-75th percentile) | 9 (8.25­9.75) | 9 (8.5-9.5) |  | 9 (8-9) | 8.5 (8-9) | 9 (NA) |  | 8 (8­9) | | 8 (7­8) | 8 (7­8) | 8 (NA) |  |
| Photo 9 |  |  | 0.992 |  |  |  | 0.892 |  | |  |  |  | 0.787 |
| Med (25th-75th percentile) | 8 (7-9) | 8 (7-9) |  | 8 (7-9) | 9 (8-9) | 9 (NA) |  | 9 (8-10) | | 8 (8-9) | 8 (8-9) | 9.5 (NA) |  |
| Photo 10 |  |  | 0.690 |  |  |  | 0.145 |  | |  |  |  | 0.054 |
| Med (25th-75th percentile) | 7 (6-8) | 7 (6.25-7.75) |  | 7 (6-9) | 8 (7-9.25) | 9 (NA) |  | 8 (7-9) | | 8 (7-8.5) | 8 (6.5-9) | 9 (NA) |  |
| Photo 11 |  |  | 0.960 |  |  |  | 0.337 |  | |  |  |  | 0.328 |
| Med (25th-75th percentile) | 5 (4.5-5.5) | 5 (4-6) |  | 7 (6-9) | 6 (5-7) | 7 (NA) |  | 7 (5.5-8) | | 6 (5-7) | 6 (5-7) | 7 (NA) |  |
| Photo 12 |  |  | 0.869 |  |  |  | 0.815 |  | |  |  |  | 0.466 |
| Med (25th-75th percentile) | 3 (2.5-3.5) | 3 (2-4) |  | 3 (2-3.5) | 3 (2-3.25) | 3 (NA) |  | 3 (2-3.5) | | 3 (2-3) | 3 (1.5-4) | 4 (NA) |  |

**Mann-Whitney U test for gender comparisons; +Kruskal-Wallis test for age and experience comparisons.*

**Supplementary Table 7.** Pairwise comparisons between the study groups.

| **Photo** | **Comparisons** | ***p^1^*** |
| --- | --- | --- |
| **Master** | Periodontists vs Orthodontists | <0.001* |
|  | Orthodontists vs GP Dentists | 0.001* |
|  | Orthodontists vs Prosthodontists | 0.041* |
|  | Orthodontists vs Laypeople | 0.002* |
| **Photo 1** | Periodontists vs Orthodontists | <0.001* |
|  | Periodontists vs Laypeople | 0.031* |
|  | Orthodontists vs GP Dentists | <0.001* |
|  | Orthodontists vs Prosthodontists | <0.001* |
|  | Orthodontists vs Laypeople | <0.001* |
|  | Prosthodontists vs Laypeople | 0.001* |
| **Photo 2** | Laypeople vs GP Dentists | 0.002* |
|  | Periodontists vs Orthodontists | 0.009* |
|  | Periodontists vs Laypeople | 0.001* |
|  | Orthodontists vs GP Dentists | 0.004* |
|  | Orthodontists vs Prosthodontists | 0.030* |
|  | Orthodontists vs Laypeople | <0.001* |
|  | Prosthodontists vs Laypeople | <0.001* |
| **Photo 3** | Laypeople vs GP Dentists | <0.001* |
|  | Periodontists vs Orthodontists | 0.010* |
|  | Periodontists vs Laypeople | <0.001* |
|  | Orthodontists vs Prosthodontists | 0.020* |
|  | Orthodontists vs Laypeople | <0.001* |
|  | Prosthodontists vs Laypeople | <0.001* |
| **Photo 4** | Laypeople vs GP Dentists | <0.001* |
|  | Periodontists vs Orthodontists | 0.274 |
|  | Periodontists vs Laypeople | 0.018* |
|  | Orthodontists vs Laypeople | <0.001* |
|  | Prosthodontists vs Laypeople | 0.012* |
| **Photo 5** | Periodontists vs Orthodontists | 1.00 |
|  | Orthodontists vs Prosthodontists | 0.020* |
|  | Prosthodontists vs Laypeople | 0.047* |
| **Photo 6** | Periodontists vs Orthodontists | 1.00 |
|  | Periodontists vs Laypeople | 0.035* |
| **Photo 7** | Periodontists vs Orthodontists | 0.104 |
|  | Periodontists vs Laypeople | 0.040* |
| **Photo 8** | Periodontists vs Orthodontists | 1.00 |
| **Photo 9** | Periodontists vs Orthodontists | 1.00 |
|  | Periodontists vs Laypeople | <0.001* |
|  | Orthodontists vs Laypeople | 0.048* |
|  | Prosthodontists vs Laypeople | 0.049* |
| **Photo 10** | Periodontists vs Orthodontists | 0.005* |
| **Photo 11** | Periodontists vs GP Dentists | 0.009* |
|  | Periodontists vs Orthodontists | 0.083 |
| **Photo 12** | Laypeople vs GP Dentists | 0.004* |
|  | Periodontists vs GP Dentists | <0.001* |
|  | Periodontists vs Orthodontists | 0.076 |
|  | Prosthodontists vs GP Dentists | 0.008* |

^1^ Only pairwise comparisons for which statistical analysis was applicable are presented.
